# Opportunistic Network Algorithms for Internet Traffic Offloading in Music Festival Scenarios

**DOI:** 10.3390/s21103315

**Published:** 2021-05-11

**Authors:** Aida-Ștefania Manole, Radu-Ioan Ciobanu, Ciprian Dobre, Raluca Purnichescu-Purtan

**Affiliations:** 1 Faculty of Automatic Control and Computers, University Politehnica of Bucharest, 060042 Bucharest, Romania; aida.manole@stud.acs.upb.ro (A.-Ș.M.); ciprian.dobre@upb.ro (C.D.); 2 National Institute for Research and Development in Informatics, 060042 Bucharest, Romania; 3 Department of Mathematical Methods and Models, University Politehnica of Bucharest, 060042 Bucharest, Romania; raluca.purtan@mathem.pub.ro

**Keywords:** mobility model, opportunistic networks, social, festival

## Abstract

Constant Internet connectivity has become a necessity in our lives. Hence, music festival organizers allocate part of their budget for temporary Wi-Fi equipment in order to sustain the high network traffic generated in such a small geographical area, but this naturally leads to high costs that need to be decreased. Thus, in this paper, we propose a solution that can help offload some of that traffic to an opportunistic network created with the attendees’ smartphones, therefore minimizing the costs of the temporary network infrastructure. Using a music festival-based mobility model that we propose and analyze, we introduce two routing algorithms which can enable end-to-end message delivery between participants. The key factors for high performance are social metrics and limiting the number of message copies at any given time. We show that the proposed solutions are able to offer high delivery rates and low delivery delays for various scenarios at a music festival.

## 1. Introduction

The study of opportunistic networks (ONs) and their applications has gained popularity in recent years, due to the increased capabilities of smartphones, which can facilitate the deployment of such networks. Opportunistic networks are derived from mobile ad hoc networks and rely on device mobility and device-to-device communication to form dynamic networks for content sharing or end-to-end message delivery. An opportunistic network routing algorithm is the logic that governs each device-to-device interaction and could be described as a set of decisions that determine which data are to be exchanged between the two devices in contact. The decision process aims to determine if the encountered device has a better chance of delivering a message than the carrier, based on local information.

This type of network is suitable for a diverse range of situations where the use of classic network infrastructure is not possible or may fail. For instance, ZebraNet [1] is a project that uses an opportunistic network to track the behavior of zebras and understand their interactions and migration patterns. Another example is a mobile application that disseminates information in a certain area affected by a network outage [2]. Moving further, it has been shown that, through ON-based applications, citizens can become sensors themselves by participating in ad hoc crowdsourcing [3].

Internet connectivity at music festivals is a real and still valid problem nowadays, since the rise of the smartphone has reshaped the way we experience entertainment events. Slow Internet speed is mostly due to large audiences gathered in relatively small geographical areas that can generate sudden demands on the network infrastructure. For festival planners, providing their attendees with a reliable Internet connection is an effective marketing strategy. As the vast majority of devices have to be associated with a base station before being able to initiate any form of communication requiring the use of a cellular network, sharing the bandwidth with thousands of devices can easily result in poor connectivity. Although event organizers can opt for the help of companies that specialize in deploying temporary Wi-Fi infrastructure equipment, this is a rather expensive solution. For example, Trade Show Internet rents a product named 4G Mega Internet Kit & Wi-Fi Hotspot (https://tradeshowinternet.com/services/4g-mega-internet-kit, accessed on 10 May 2021), which can support up to 300 devices for 2650$ per day, (this applies only in the US, for up to 12.5 GB of data daily).

Our focus is to find a solution in the form of an opportunistic algorithm that can complement the already existing network infrastructure. The end goal would be to reduce the cost of additional network infrastructure equipment necessary at music festivals by taking advantage of the communication capabilities of the festival goers’ devices. For this, we envision a situation where a group of friends come together at a festival and get separated at some point. Normally, if there is a large crowd at the festival, chances are that they will have issues trying to communicate with each other using a Wi-Fi access point or mobile broadband. We want to help in designing a solution where this problem might be alleviated, by offering users opportunistic-based delayed communication through the smartphones of the festival participants. Thus, the main objectives of this paper are to:

- Design a mobility model which can emulate the movement patterns of festival attendees and use it to test various opportunistic algorithms;
- Study already existing opportunistic routing solutions;
- Identify the key parameters that have the potential to increase the performance of an opportunistic algorithm in a music festival scenario;
- Create an opportunistic algorithm specifically tailored for high performance at crowded events;
- Study the impact of Bluetooth and Wi-Fi Direct on algorithm performance.

Through experimental analysis, we identify three opportunistic algorithms that can work best at a music festival, namely binary Spray-and-Wait [4] and two custom-designed algorithms that we propose and present in Section 4. We test the solutions in an opportunistic network simulator using the traces generated by our mobility model, designed to mimic a festival crowd. The node movement is driven by social relationships or the node’s interest in a particular area on the map (e.g., food court).

The rest of the paper is structured as follows: Section 2 provides an overview of opportunistic networks, then presents two case studies: a music festival in Belgium and a street festival in Zurich. The proposed festival mobility model is presented in Section 3, while, in Section 4, we propose two opportunistic algorithm for a festival scenario. The test data and results are analyzed in Section 5. Finally, Section 6 outlines the main conclusions and identifies recommendations for further research.

## 2. Background and Related Work

This section introduces the idea of opportunistic networks and outlines their main characteristics, then it illustrates these concepts at work in two real-world scenarios, a festival in Belgium, and one in Zurich.

### 2.1. Opportunistic Networks

Opportunistic networks have evolved from MANETs (mobile ad hoc networks), which have the significant drawback that they store routing information and update frequently. On the other hand, opportunistic networks are dynamically built when mobile devices collaborate to form communication paths while users are in close proximity, without requiring state information such as routing tables, being instead based on a store-carry-and-forward paradigm. ONs are a type of delay-tolerant network (DTN). DTNs are composed of independent internets with connectivity inside the networks, but only occasional connectivity between them. They consist of DTN regions and DTN gateways. While DTN gateways bridge together DTN regions that may operate on different protocol stacks, ONs are more flexible environments.

To illustrate the flexibility of ONs, here are their main characteristics:

- Nodes can communicate with each other, although there is no route between them;
- Nodes do not require information about the network topology in order to be able to communicate;
- The routes between nodes are built dynamically
- any node can be used as the next hop for a message, if it may bring the message closer to the destination;
- Since the paths between nodes are assumed to be dynamic, this aspect may increase the delivery delay for a message;
- Each node acts as a gateway for a message, exploiting its local knowledge at that time in order to determine the best next hop.

An opportunistic protocol is an algorithm that operates under the constraints just mentioned. This protocol may try to achieve end-to-end message delivery or to disseminate information into the network based on the interests of the nodes and their social relationships.

ONs apply to many real-life scenarios such as wildlife tracking [1], sensor networks [5], military networks [6], intermittent Internet connectivity [7], crowd management [8], emergency crises [9], etc.

Large mass events pose a tremendous load on the classic network infrastructure due to the large number of devices concentrated in a relatively small geographical area. Where the traditional means of communication may fail, ONs come to light. The next two sections will discuss applying ON routing protocols at festivals that fall under the umbrella of large mass events.

### 2.2. Belgium Festival Study

The aim of [10] is to propose suitable routing algorithms to be used at mass events for delivering small messages through opportunistic communication. The researchers used GPS traces recorded at a festival in Belgium to test their ideas in a real-world scenario.

ONE (Opportunistic Network Environment, http://akeranen.github.io/the-one/, accessed on 10 May 2021) was used as a test environment. From 5300 unique devices detected each day of the festival, 1000 were selected in the test scenario. The conclusions obtained by the authors were that Epidemic [11] and binary Spray-and-Wait [4] are the most suitable protocols to be used at mass events. Epidemic is most reliable in an emergency situation, when the network contention and power consumption are not taken into consideration, but Spray-and-Wait outperforms Epidemic in an energy-conscious environment. Both protocols require a small number of control messages, while achieving better values for delivery delay and delivery ratio than more complex protocols (e.g., PRoPHET [12]).

### 2.3. Zurich Festival Study

In [2], the authors propose an opportunistic protocol based on the Wi-Fi hotspot capability of smartphones. The researchers have built an application that measures some crowd parameters at mass events (e.g., crowd density) and allows the propagation of messages by switching the role of a device between client mode and access point mode. Relying the entire strategy on the Wi-Fi hotspot functionality of smartphones was a choice motivated by compatibility reasons, as this feature is considered universal in devices nowadays. An essential aspect of this work was considering the human body to have an impact on the Wi-Fi signal strength.

NetLogo (https://ccl.northwestern.edu/netlogo/, accessed on 10 May 2021) was used as a test environment and was configured using real data collected during the Zurich festival. It is worth mentioning that the Zurich festival is a street festival, so the mobility pattern may differ from that of a music festival in terms of the average standing time for a participant and the time between standing and walking.

Test results showed that, for two clients per access point and with 40% of the nodes assuming the access point role, a message reached all 1000 users in around 33 min. For a ratio of 20–25% nodes acting as access points at any given time, the convergence time value was 40 min. The conclusion was that Wi-Fi hotspot technology is a viable option for message propagation at large-scale events, while carefully considering the role switching parameters and crowd dynamics.

## 3. A Festival Mobility Model

In this section, we analyze a festival dataset that will be utilized for our solution, and then we discuss which protocols would be suitable as a means of communication for ONs and their limitations. We also discuss a type of application that might benefit from the use of ONs and what communication patterns it implies. We then analyze existing mobility models and their drawbacks, and propose a novel mobility model better suited for approximating festival contacts.

### 3.1. The Sonar Festival Dataset

#### 3.1.1. Description

The Sonar Festival dataset [13] offers information about the mobility of participants at the Sonar festival in Barcelona, Spain, which took place on 18–20 June 2015. This festival is a multistage event with more than 100,000 attendees in two main venues, Sonar by Day and Sonar by Night. The data were collected during the daytime in six locations of the venue and have been anonymized in order to reduce privacy issues.

The data consist of time points recorded by Raspberry Pi 2 nodes distributed in strategic points across the festival grounds, in order to maximize the covered area. Around six million Wi-Fi events were recorded during this experiment.

#### 3.1.2. Challenges

For validating our proposed solution, we used MobEmu [14], a mobile interaction tool that is able to simulate the behavior of mobile nodes and the way they interact with each other. For this reason, the Sonar festival trace data had to suffer some transformations. Some of the filters applied were:

- Duplicate filter—we had to delete the duplicates from the dataset, caused by clock synchronization errors and buffering between the server and the scanners;
- Format filter—we had to convert the anonymized MAC address to a node identifier format supported by the simulator; the end goal of parsing the dataset was to obtain contacts between nodes, which are the centerpiece structures of a mobility trace; a contact is an interaction between two nodes, which has a start and end timestamp and during which messages are exchanged following a communication protocol;
- Time filter—we checked the data for outlier values and validated that all timestamps were between 9:00 a.m. and 12:00 a.m.

Looking at the data, we noticed some anomalies:

- Some of the timestamps were between 12:00 a.m. and 9:00 a.m., although the researchers mention they had only collected data between 12:00 p.m. and 10:00 p.m.; we chose not to include data recorded during the night in the final dataset;
- Some of the nodes were observed consistently in the same place for hours; these data points might exist because the main stage was surrounded by food trucks and other non-music related attractions; naturally, if a node spent around 11 h at a location, it can be assumed that it was the phone (or other device with Wi-Fi connectivity) of one of the staff members;
- Some of the nodes were observed for a brief period of time in a location, so we discarded them.

After computing the time a node spent at the festival each day, the following decisions were reached:

- Each day, there were a significant number of nodes present at the festival for less than 60 min; whatever the causes might be, we have chosen not to consider these nodes as contact-worthy (in order to account for communication or data collection errors that might affect and alter our conclusions);
- Although there were around 100 nodes that spent more than 10 h at the festival each day, we considered them for contact opportunities only if that time was not spent at the same location.

**Figure 1:**
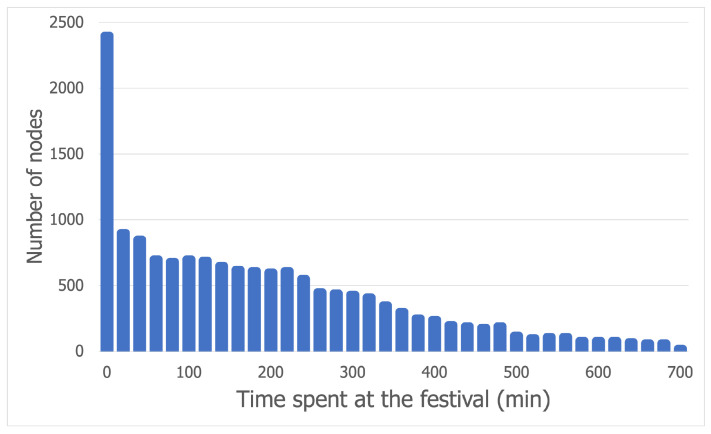
Outlier values for day 1 of the Sonar dataset.

The previous conclusions are supported by Figure 1, which shows the outlier values for the times spent by a node at the festival on the first day (the charts for the second and third days are very similar).

#### 3.1.3. Analysis

In order to understand the mobility of people during a festival, it is crucial to perform an analysis of the current dataset. The conclusions of this analysis will provide us with more in-depth knowledge about the mobility patterns and will help us later replicate this behavior with a synthetic mobility model. The following histograms will offer some insights into the Sonar festival dataset. For this analysis, only data from the first day of the festival will be discussed, but the conclusions hold for the other two days.

**Figure 2:**
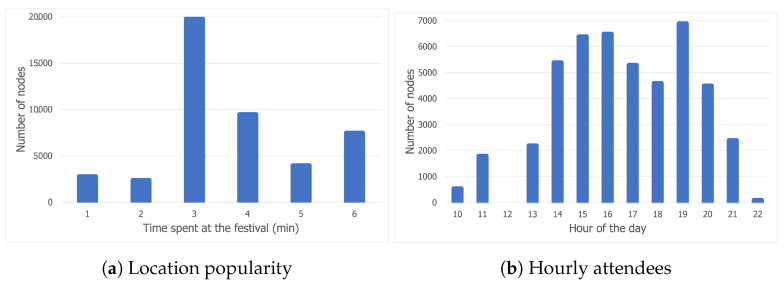
Location popularity and hourly attendees for day 1 of the Sonar dataset.

Figure 2a displays the distribution of nodes across all six locations at the festival venue. The most popular location is 3, with about 20,000 nodes being detected during the day. Although the number of people attending the first day of the festival was 9616 after blacklisting some nodes, a person can show up multiple times at a certain location throughout the day, which explains why approximately 20,000 nodes were present at location 3. Locations 4 and 6 displayed a similar level of popularity, reaching figures of slightly under 10,000 and 7500, respectively. The least popular locations were 5, 1, and 2.

Figure 2b shows the number of attendees during every hour of the first day of the festival. The number of people attending the festival steadily increases, starting with 2:00 p.m. until 5:00 p.m., when there is a slight drop. It can be assumed that the main event started at 7:00 p.m., given the fact that the number of attendees reached a peak at that time. The chart follows a downward trend after 8:00 p.m., with the least number of people being recorded after 10:00 p.m.

#### 3.1.4. Generating Contacts

The primary purpose behind processing and analyzing this dataset is to manage to generate contacts with the extracted information. Unfortunately, this dataset lacks an essential piece of information: it is impossible to infer the location of a node. Due to the way data were collected (using Wi-Fi scanners which would cover a large area), it is unfeasible to determine the exact position of a node or its neighbors. Although the original dataset contains information about the signal strength, it is not available to the public. Consequently, we have decided to create a synthetic mobility model, which will be discussed thoroughly in Section 3.4.

#### 3.1.5. Conclusions

Even though this dataset has not proved to be a trace to use in a simulation, it still holds some valuable insights about the mobility exhibited by festival attendees:

- People spend different amounts of time at a festival, which could be implemented in a synthetic mobility model;
- Some areas are more popular than others, and hence more crowded;
- The number of people varies throughout the day.

### 3.2. Wi-Fi Direct vs. Bluetooth

Choosing one technology over the other implies making different sacrifices. Although Bluetooth has a better power consumption, this comes at the cost of a lower transfer speed. Moreover, Wi-Fi Direct has a larger range of transmission given optimal conditions, but the crowd present at a mass event such as a festival is far from optimal.

Wi-Fi experiences a considerable drop in performance in the context of crowded events [2]. The authors have named this phenomenon “the effect of dense crowds”. They state that, because the Wi-Fi signals are mainly transmitted in the 2.4 GHz range (which happens to be around the resonance frequency of water), people might affect the transmission of these signals. Experiments have established that the human body can attenuate the signal, and the authors’ conclusions were that the Wi-Fi signal strength loss caused by the human body is stronger when a person is closer to an access point than further away [15].

Furthermore, the Wi-Fi signal drops when receiving the signal from behind a person than receiving it in front, and smartphones carried inside trouser pockets are still able to communicate over a distance of up to 50 m, but they can sporadically lose their connection when the subjects move further away [2]. Although these tests were not performed on Bluetooth, it is worth taking into account that Bluetooth also operates in the 2.4 GHz range, so there may also be some attenuation of signal in this case.

In light of these findings, the maximum ranges should be set lower than their current values. It is safe to assume a range of 5 m for Bluetooth devices and 30 m for Wi-Fi Direct devices. Although MobEmu nodes do not run over an actual network stack implementation, the comparison between Bluetooth and Wi-Fi Direct can be useful when generating contacts between people in a crowd. For the simulation to be more realistic, when using Bluetooth as a means of communication, a node should have an average of 5–7 contacts at a certain point in time, while Wi-Fi Direct allows for a higher number of contacts within a larger radius.

### 3.3. Communication Use Case at a Music Festival

When analyzing the mobility patterns that people attending a festival follow, we can observe that, during a concert, very few nodes change their position. Furthermore, when not attending a concert, people usually move from a stage to another, or go to the food court or other entertainment areas. We chose to focus on the former scenario because it poses a more significant challenge, since that is when the network is heavily loaded and barely works, resulting in poor connectivity and large delivery delays for festival attendees.

First of all, in order to create our festival mobility model, we need to address the way messages are generated during a simulation. To do so, we need to identify what applications would be relevant to the people attending a music festival. We propose such an application: a messaging app similar to WhatsApp, which could benefit from an opportunistic network through the use of an API.

In this use case, in terms of the communication pattern employed, participating nodes are more likely to send messages to the nodes in their social network than to any other nodes. Our proposal for the default method of generating messages during a simulation run using MobEmu is the following:

- Pick a generation time—-this is done randomly, since a festival participant might need to communicate with other people at any time;
- For each node in the simulation, generate a fixed number of messages and copies corresponding to a message—the destination type of a message is established through the use of a Zipf distribution (A method for generating Zipfian random values is presented here: https://medium.com/@jasoncrease/zipf-54912d5651cc, accessed on 10 May 2021); the nodes which are both in the social network and the discovered community (A node’s discovered community is a group of other nodes that it comes in contact with often. Aside from the group of friends (which will also be part of the social network community), a discovered community will also most likely include “familiar strangers”, meaning unknown persons that are often encountered for long periods of time.) of the node are more likely to be selected as the destination, followed by nodes in the social network, nodes in the community, and finally random nodes.

We had to modify the default method in order to better serve the communication pattern described earlier. First, we need to establish the concept of “chat pairs”. A chat pair is a pair of two nodes which attend the festival together and exchange messages while one of them is away. This behavior is implemented in the synthetic mobility model presented in the Section 3.4. Moreover, each node has a number of friends that are eligible message destinations. The messages between chat pair nodes are generated more often than the messages between regular nodes, every 6 and 15 min, respectively. The new method consists of the following steps:

- If the node is not part of a chat pair, pick a random message destination, the only constraint being that the destination has to be a friend;
- For every chat pair, generate two messages, each message originating from one of the two pair members.

### 3.4. Simulating Festival Mobility Behavior

Before attempting to propose our own festival mobility, we analyzed existing solutions. One potential solution was PedSim (https://github.com/chgloor/pedsim, accessed on 10 May 2021) in conjunction with the ONE simulator (https://akeranen.github.io/the-one/, accessed on 10 May 2021), used in [16] to simulate pedestrian mobility in an open square and a subway station. However, when comparing the existing solutions with the real-life traces that we analyzed (such as the Sonar Festival dataset), we found that the accuracy of PedSim was not sufficiently high. We wanted to have a better approximation of real movement at a festival, which is why we opted for creating our own mobility model, which we present here.

After conducting a thorough analysis of a real dataset recorded at the Sonar festival in Spain, we have concluded that it is best to create our own novel generator of contacts due to a couple of reasons:

- Even after applying outlier removal to the Sonar dataset and grouping the nodes by location, we found it challenging to generate a close-to-reality trace due to the lack of location information; without any data regarding the vicinity of a node or its social ties, no strategy other than randomly generating contacts can be assumed to be better;
- The validation of an opportunistic network algorithm relies heavily on how realistic the movement models used in the simulation are [17]; therefore, it is of paramount importance to identify realistic mobility models in order to tailor an algorithm for the desired scenario and to later fine-tune it for better performance.

One can test an opportunistic algorithm in two ways, namely by using real mobility traces or a synthetic model. Both strategies come with their limitations:

- CRAWDAD (https://crawdad.org/, accessed on 10 May 2021) is an archive of wireless trace data publicly available to the research community; unfortunately, these traces are related to particular scenarios and can hardly be generalized for different use cases; moreover, there is only one festival trace available, but the data recorded did not suit our needs;
- Many mobility models are based on the random movement of individuals such as the Random Walk model (where nodes move by randomly choosing a direction and speed) or the Random Waypoint model (where pauses are introduced between changes in direction or speed); however, there are also two mobility models, CMM [17] and HCMM [18], which are founded on social network theory and, therefore, closer to reality.

The Community-based Mobility Model [17] (HCMM) is based on the observation that opportunistic networks consisting of mobile devices display mobility patterns based on human decisions and social behavior. Furthermore, it is crucial to understand that individuals move in groups and between groups. It is comprised of three steps: modeling social relationships, identifying communities, and groups in the networks based on step 1, and using an algorithm for the dynamics of the nodes based on social relationship strength.

The representation of social relationships is done with the use of a weighted graph that models the strength of the interactions between nodes. CMM uses a symmetric matrix called the interaction matrix, which contains information about the social degree of interaction between nodes, and is used to generate a connectivity matrix necessary for the second step. At step 2, a community detection algorithm is executed, which results in a network composed of different groups of nodes. After this process, each of the identified communities is randomly associated with a specific grid location.

The third step establishes how the nodes move across the simulation space. This is achieved by assigning a goal to each node. A goal is simply a cell on the simulation grid, which acts as a final destination, and it is selected by computing a metric called social attractivity for every grid cell and choosing the one with the highest value. This metric is measured as the strength of social relationships with the nodes within that cell. For this model to work, a social network is required as an input.

This Home-cell Community-based Mobility Model [18] (HCMM) takes the CMM model one step further and introduces the idea that users are attracted by particular physical locations [18], in which they tend to preferentially spend their time, such as the workplace, their home after work, etc.

CMM and HCMM serve as a great inspiration when implementing a mobility model. HCMM was the mobility model already implemented in the simulator used for this project, MobEmu. The shortcomings which led to a custom implementation of a mobility model, with the ideas of HCMM as a starting point, were that HCMM:

- Has no support for a custom social network;
- Measures cell attractivity differently than CMM—the social attraction towards a cell is evaluated based on the social relationships with nodes having that cell assigned as their home cell; the idea of a home cell is not relevant in a festival scenario, but it is of great importance when simulating a workday in a city;
- Allows for a single group to be placed in a cell regardless of cell size;
- Has no crowd density mechanism.

### 3.5. A Proposed Festival Mobility Model (FMM)

**Figure 3:**
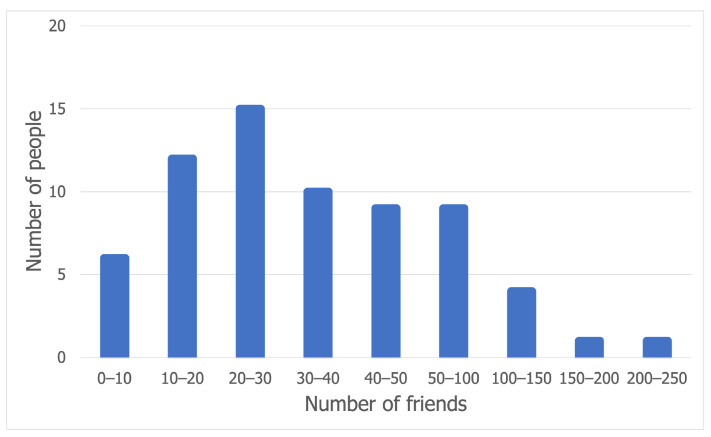
Number of Facebook friends attending a festival.

The model that we propose here borrows the concept of an interaction matrix from CMM. The matrix can also be derived from social investigation, besides generating it with social network models. In our case, this social information, which shows how many of a person’s Facebook friends have attended a festival, was gathered using a form with 67 valid answers. The survey asked participants what festivals they attended in the last year and, through Facebook check-ins, which of their social network friends also participated. The results of the survey show that the majority of people participating in this study have between 3 and around 50 Facebook friends that attended the same festival as them, as can be observed in Figure 3.

The information provided by this form has been used as a pool of values when assigning the number of friends to a node. The weights of the interaction matrix have been generated randomly, but making sure a node is part of a custom-sized community. The second step of CMM has been skipped and the communities have been established along with the number of friends per node. It was assumed that a person attending a festival has around five close friends who form its community.

The reason behind choosing 5 as the average community size is the result of a study on human social behavior at large-scale events [19]. The event where the experiment took place was an 8-day 6-stage music festival with 130,000 attendees. A part of this research was to model the crowd’s internal structure with the help of a community discovery algorithm. The groups are defined as sets of people that are frequently located in the same spatio-temporal bins. The model consists of directed graphs, with the weight of an edge being defined as “the number of co-occurrences of participant *A* with participant *B* divided by total number of occurrences of participant *A*” [19]. For an edge to be accepted as valid, a constraint was imposed, which specifies that the only links chosen were the ones that occurred in more than two different locations, with a weight of at least 0.5 [19]. After employing a rewiring algorithm [20] and performing 35 tests, the conclusions were that the average group size was 5 (with a standard deviation of 4.32) and the average number of links for a node was 3 (with a standard deviation of 2.60). A rewiring algorithm is the process of creating a randomly generated network from an existing network while preserving some of its topological properties, such as the degree of nodes, so one can focus only on certain aspects of the network design instead of dealing with the whole complexity of the system.

The groups are assigned randomly to a cell in the grid, but not without taking into account the density of the crowd. A crowd safety and risk analysis study [15] conducted in 2019 shows that the upper limit for standing/viewing spaces is 5 people per square meter. The study also maps different densities of people per square meter. For this model, 4 ppsm (people per square meter) has been chosen as the upper limit for the most crowded areas at a festival, usually registered near the stage. 3 ppsm has been chosen as an intermediary density, as the distance to the stage increases. 2 ppsm will be the minimum density, used for the back rows of a crowd.

Another shortcoming of the implementation of HCMM, which emerges from the fact that a cell can host only one community, is that the members of that community are randomly placed in that cell, sometimes at distances that would not comply with a festival crowd distribution.

One important aspect of our proposed festival mobility model is the way it handles crowd distribution. First of all, depending on the position of the stage (assumed to be in the north of the grid), the model computes a maximum number of hosts per cell based on the three values for crowd density mentioned earlier. Moreover, it randomly chooses a cell for a group, without exceeding the maximum number of hosts allowed in that cell. Lastly, it generates coordinates for every host in a group. This process is done randomly, but only after further partitioning the cell, so the nodes of a group do not appear scattered across the cell. Depending on the dimensions of the grid, the area of the cell can be divided by its width or by its height. The model randomly places a maximum of two groups in the same partition, after the division of the cell area.

**Figure 4:**
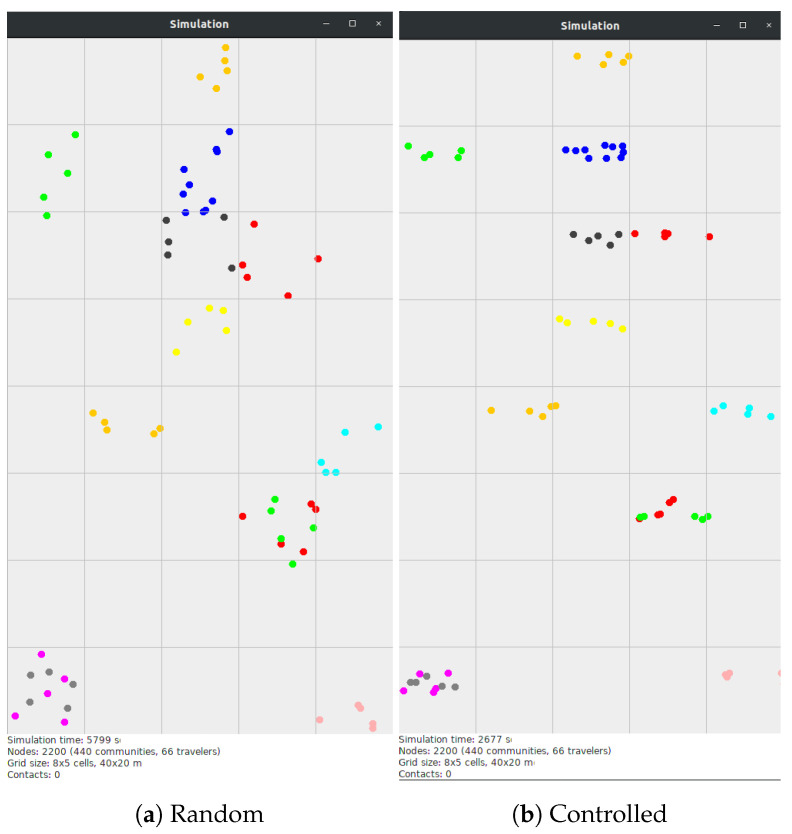
Crowd distribution in FMM (nodes with the same color belong to the same community).

The results of this approach can be noticed in Figure 4, which shows screenshots captured from the MobEmu simulator, where nodes with the same color are part of the same community. Figure 4a displays community placement without partitioning the cell area. Some of the groups appear to be composed of nodes far away from each other, which is very unlikely in a festival crowd. Figure 4b, which shows the case where the partitioning mechanism is employed, looks more similar to a real crowd distribution.

The model keeps the CMM and HCMM node categories, i.e., hosts and travelers. The hosts are the nodes belonging to a custom-sized community and might play the role of festival attendees, while the travelers are independent nodes, community-free, which might play the role of festival staff.

In terms of movement, a node can move by itself or can move along with its community. Given this behavior, three types of movement are distinguishable:

- Move alone towards a target destination;
- Move with the whole group towards a target destination;
- Return to the community after having spent a certain time at the destination.

FMM has been configured to perform the first type of movement three times more often than moving an entire community with the help of a Zipf distribution of size 4. The reason behind this choice is that, during a short simulation (30 min–1 h), it is more likely that a festival attendee will move alone to the food court or the restroom area and then get back to the group rather than an entire group relocating.

As for the target destinations which drive the movement, we have identified two main categories: edge cells (assumed to be the food court area or restroom area) and friend cells (where determining the friend cells of a node is a process very similar to the one employed by CMM). For a given node, the process consists of the following steps:

- For every cell on the grid, compute a metric called cell attractivity, defined as the sum of the social relationship weights of the nodes present in that cell with regards to the given node;
- Normalize the sum by the total number of nodes present in that cell.

It is only natural that a node would choose as its target a cell where more friends will be. Another aspect of node movement is the time it would take for a node wandering away from its community to get back. FMM will pick a time between two ranges depending on the type of movement. For instance, a node moving towards a friend cell will be away from the community for a time interval of 10–20 min.

Given the fact that a festival stage is a crowded place, we have chosen a threshold that measures how many nodes are moving at every time instance *t* of the simulation. This limit is configurable and is currently set at 5% of the number of nodes in the simulation.

Thus, as shown here, the proposed festival mobility model generates the movements of nodes inside a given space. Then, based on the protocol that we want to test and analyze, we consider a contact whenever two nodes are closer to each other than the range of the short-distance protocol employed. The mobility model is therefore decoupled from the actual routing algorithm implementation, which comes on top of the contacts generated by the model. At each contact (per the model), the routing decision function is run on the two encountering nodes, thus deciding which messages are exchanged.

We performed a validation of our model using the Sonar festival dataset, in terms of similarity between number and duration of contacts, interactions, etc., which we wish to present in future work. We would also like to extend this model to be able to accommodate more stages, to implement conclusions found in real festival traces, and to support more types of node movement.

## 4. An Opportunistic Routing Protocol for a Festival Scenario

In this section, we propose and present two opportunistic routing protocols especially designed for music festivals. Our aim is to determine a routing protocol which will best suit the communication patterns at a music festival. We formulate the following qualities of such an algorithm:

- Ideally, it should consume a small amount of resources;
- The delivery delay should be less than 5–10 min for an acceptable quality of service;
- The delivered messages ratio should be above 90%.

In Section 3, we pointed out that a festival crowd mimics a social structure. The natural behavior of attendees is to share these collective experiences with a group of friends. Thus, we argue that a routing algorithm could benefit from taking into consideration the social dimension when deciding the next hop for a message.

To make our approach more resource-efficient, we have decided to use the idea proposed by the Spray-and-Wait algorithm [4], which bounds the number of copies a message can have at any given time. By limiting the number of copies, a flood-based forwarding scheme is avoided. Moreover, we base the forwarding decision on two social factors, namely node centrality and the number of common friends.

Node centrality was first proposed by the algorithm Bubble Rap [21] as a metric to quantify node popularity. Bubble Rap embraces the fact that because a smartphone is carried by a person, the mobility pattern exhibited by such ad hoc networks closely follows people’s mobility patterns. Consequently, the forwarding decision relies on two social metrics: centrality and community structure. Detecting the community structure is performed using the K-clique algorithm [22]. Although the MobEmu simulator has this mechanism for community detection already implemented, it is a time-consuming process.

Our algorithm measures the centrality of nodes similarly to the Degree forwarding scheme [21] proposed by Bubble Rap. Thus, centrality is defined as the number of unique encounters a node has over a period of time. Besides being an indicator of node popularity, centrality can also be used to depict the mobility degree of a node. During a concert, most people remain static, while some may go to the food court and drinks area or briefly join another group of friends. In the case of movement, such a person will make contact with other nodes and become a better candidate to carry a message from one place to another.

The number of common friends is another valuable social metric we have chosen as a substitute for community detection. Given the fact that the number of common friends is public information on Facebook, with the appropriate permissions from the owning company and the festival attendees, it could be easily collected. For this to work, each device should store an offline list of Facebook (or other social network) friends that can be securely exchanged with an encountered node at the beginning of a contact. This way, a node would not depend on global information when encountering a node, only what it has stored locally and what the encountered node advertises.

We favor the number of common friends over K-clique community detection due to the complexity of the two processes. Retrieving the number of common friends has a complexity of O(1), whilst detecting the community structure can have a worst-case complexity of $O(n^{2})$ if we use a global approach, or O(n) if we use a local approach when computing the familiarity set of a node. The familiarity set is the set of nodes which have been in contact with a node for a period which exceeds a certain threshold. In terms of graph theory, there is an undirected edge between the nodes which have fulfilled that criteria. In the worst case scenario, a node keeps track of the familiarity sets of all encountered nodes.

The centerpiece idea is that we prioritize nodes with higher centrality values or nodes that have more common friends with the destination, giving them more message copies to spread on their own, whilst we give just one copy of the message to the other nodes. In other words, we combine the two versions of the spray phase of the algorithm Spray-and-Wait (SW) [4]. We perform the binary version (give away half of the copies) for nodes that are likely to spread the message over a bigger area (high-centrality nodes) or for nodes that are more socially connected to the destination (more friends in common). With regard to the rest of the nodes, they receive only one copy (as in the source version of Spray-and-Wait) and proceed to the wait phase. The wait phase implies forwarding the message only to the destination node.

We have designed two different algorithms based on these observations: Social Spray-and-Wait 1 (SSW1) and Social Spray-and-Wait 2 (SSW2). The former has a more strict forwarding decision: a node gives away half of a message’s copies if the encountered node has superior values for centrality or the number of common friends. This prevents a node from forwarding half of the number of copies to another node that is too similar to itself. To overcome this potential issue, a centrality threshold was introduced in SSW2. We present the two proposed solutions in Algorithms 1 and 2.
**Algorithm 1:** SSW1 forwarding.
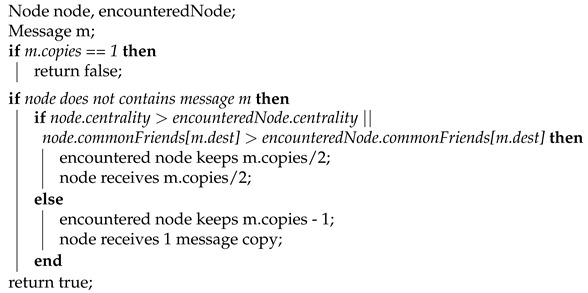

**Algorithm 2:** SSW2 forwarding.
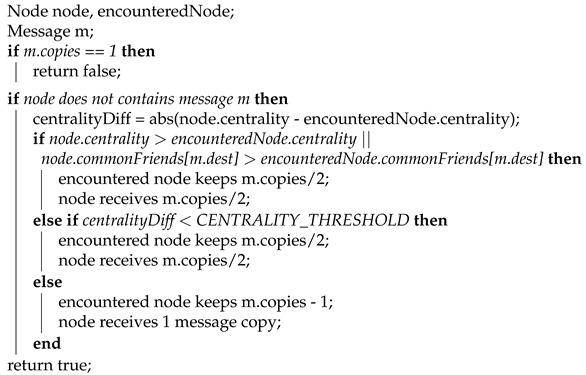


It should be noted here that, although in Section 5 we evaluate the viability of Bluetooth and Wi-Fi Direct as means of communication in a festival scenario, they have certain limitations that would need to be addressed. The main issue is that, in normal functioning and most cases, both protocols require pairing, which would not be easily done in a large scenario. Furthermore, high congestion may be caused by many devices communicating wirelessly at the same time, as shown in [23]. However, the solution we envision would also include a component that can allow a node to actively communicate on a wireless interface only at certain times, as we have shown in [24]. This would reduce the congestion and the observed node density. Furthermore, such a scenario would benefit from other orthogonal solutions, such as having edge nodes that could take some of the load, or other similar devices. This is an interesting topic and something that we intend to further pursue in the future.

## 5. Evaluation

In this section, we evaluate the performance of the opportunistic algorithms proposed and presented in Section 4. We first introduce and explain the metrics used to measure performance and then we focus on the characteristics of the mobility traces that we used for evaluation. In the end, we compare and contrast our two algorithms to other existing solutions, and focus on the most suitable design for a music festival.

### 5.1. Algorithms and Metrics

In order to highlight the benefits of our proposed solutions in a festival environment, we compared them with several well-known algorithms previously proposed: Epidemic [11], Spray-and-Wait [4], Spray-and-Focus [25], and Bubble Rap [21].

The Epidemic protocol operates on a flooding-based scheme. A node exchanges messages with the other nodes in range. The only constraint is that, if a node already has a specific message, it is not duplicated. On the other hand, Spray-and-Wait (SW) bounds the number of message copies allowed to exist at a given time. It has two stages: if the number of copies is greater than 1, a carrier forwards only a part of the message copies to encountered nodes; if there is only one message copy left, the carrier waits to encounter the destination. The first stage can be accomplished in two ways: forward a single copy (the source version of the algorithm) or half of the message copies (binary version) to an encountered node. The binary and source versions of the algorithm will be abbreviated as SWb and SWs, respectively.

Spray-and-Focus combines the binary version of the spray phase of SW with a utility-based forwarding decision. The utility function replaces the wait phase of SW and uses last-encounter timers in order to decide whether to relay a message or not.

As was already discussed previously, the Bubble Rap forwarding decision relies on two social metrics: centrality and community structure. Nodes with higher centrality or community members of the message destination will be selected as relays. It is worth mentioning that we have tested Bubble Rap without having nodes store and update a local approximation of each encountered node’s familiarity set due to high RAM usage. As a result, community detection is performed based on contact duration and a node’s local familiarity set.

Besides the opportunistic routing protocols proposed and presented in Section 4, we have also tested different combinations of the same ideas, in order to see the differences in performance. There were four criteria which could be applied at different stages of the algorithm (with Table 1, describing the actions performed during each phase of the forwarding decision):

- Forwarding criterion 1 (FC1)—forward the message if the encountered node is a friend on a social network with the message destination and the carrier node is not;
- Forwarding criterion 2 (FC2)—forward the message if the encountered node has more friends in common with the message destination;
- Forwarding criterion 3 (FC3)—forward the message if the encountered node has a higher centrality;
- Forwarding criterion 4 (FC4)—forward the message if the centrality difference is less than a predefined threshold.

The performance of the analyzed algorithms is established with the help of the following metrics:

- Delivery rate—the ratio between the number of delivered messages and the total number of created messages;
- Delivery delay—the time elapsed between the generation of a message and its delivery;
- Overhead—the ratio between the total number of exchanged messages and the total number of created messages;
- Number of hops—the number of nodes through which one message has been relayed until reaching its destination; this metric and the overhead are indicators of the network and node congestion.

### 5.2. Test Data

The test data consist of two traces generated in the MobEmu simulator [14] using the mobility model described in Section 3.5, customized with different parameters (as presented in the rest of this section).

#### 5.2.1. Bluetooth Trace

The first trace that we generated simulates communication via Bluetooth. The input and output parameters for this trace are displayed in Table 2 and Table 3. The number of nodes is among the output parameters because it is determined by the number of grid cells and the crowd’s density at different points on the map. For this scenario, Bluetooth was the only protocol used as means of exchanging data during contacts.

**Figure 5:**
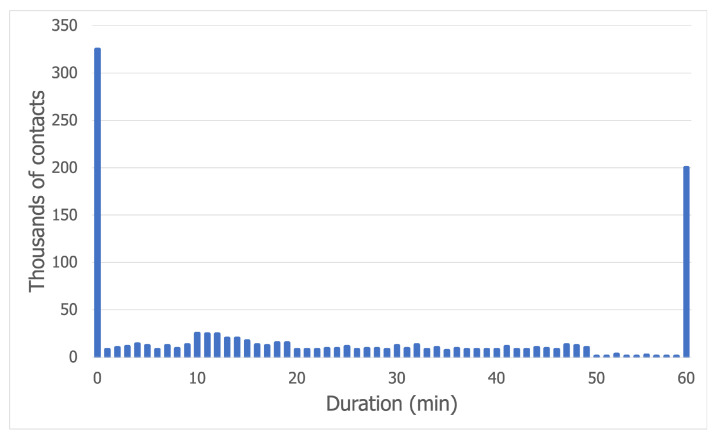
Contact duration distribution for the Bluetooth trace.

**Figure 6:**
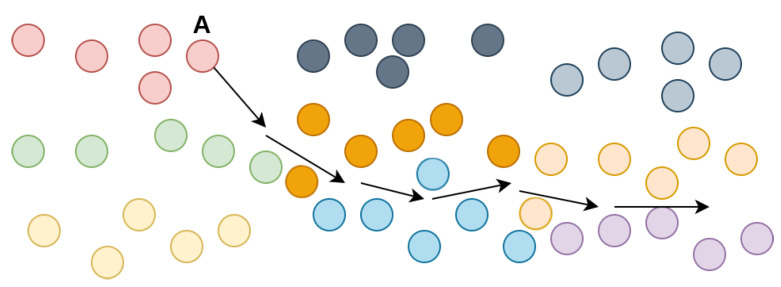
Node behavior example.

The contact duration distribution for the Bluetooth trace is displayed in Figure 5. The vast majority of contacts, slightly under 350,000, lasted less than 1 min. This trend is due to the fact that, at a concert, the crowd is very dense, so a node will encounter many people as it moves towards a target. The contacts with a duration of more than 55 min correspond to the nodes which have remained static for the entire trace time. The other values can be explained by the nature of the movement. To make this clear, we illustrate an example in Figure 6. As node A moves away from its community to a cell on the edge of the grid (assumed to be the food court and drinks area), it breaks the long contacts maintained with its community, then it creates very short-lived contacts with the nodes on its way to the edge of the map. After a while, it goes back to its community, creating other short-lived contacts as it makes its way back through the crowd.

#### 5.2.2. Wi-Fi Direct Trace

The Wi-Fi Direct trace was designed taking into consideration the protocol’s transfer speed, its radius in a dense crowd environment, and a 7-client limit per mobile access point for Android phones. It implements the following constraints, which will be later motivated by a Wi-Fi Direct analysis:

- Only nodes moving away from their community will play the role of access point (AP) or group owner (GO);
- Once a node is reunited with its community, it stops being an AP;
- to preserve their battery, AP nodes will alternate between being on and off for intervals of 5 min;
- A node can connect to an AP for 1 min;
- A node cannot connect to the same AP twice in a row;
- The rest of the nodes also exchange data via Bluetooth.

In a real-life scenario, a node could detect that it is moving away from its community by employing the following logic: among its Bluetooth peers, the number of friends is less than a certain threshold. As a node moves further away, its friends will no longer be within reach. This will trigger the node to switch from Bluetooth to Wi-Fi Direct and become an AP for the other nodes.

We have chosen to emulate this behavior due to the limited area of the simulation. The grid in the Wi-Fi Direct trace is 30 × 20 m^2^, while the Wi-Fi Direct radius has been set at a maximum of 30 m. Hence, an AP node will cover the entire space. If we were to allow all the nodes to form Wi-Fi Direct groups in such a restricted area, our results would not be relevant. The end goal was to see what happens in a less interconnected environment.

The Wi-Fi Direct architecture, built upon the infrastructure mode defined by the IEEE 802.11 standard, specifies two roles: a P2P group owner (similar to the role of an AP in a classic infrastructure) and a P2P client, which can act as a legacy station and become a client to an AP/GO, while simultaneously playing the role of a GO. The terms AP and GO will be used interchangeably, as they both refer to the same concept.

P2P groups can be regarded as Wi-Fi infrastructure networks. The standard way of establishing a group is comprised of multiple stages:

- The P2P devices perform a classic Wi-Fi scan, which may lead to discovering other Wi-Fi networks and P2P groups;
- A process called device discovery happens afterward; the devices alternate between two states for randomly distributed periods: search state and listen state; during the search state, a device sends probe requests on channels 1, 6, and 11 in the 2.4 Ghz frequency band; in the listen state, a device will listen for probe requests and then send probe responses;
- GO negotiation will be initiated after two devices have discovered each other; the GO will be elected as a result of a three-way handshake, after which the devices will exchange their GO intent values.

Should all the nodes be Wi-Fi Direct-enabled, they would perform the device discovery process, negotiate group ownership and form a highly interconnected network, a scenario we would like to avoid. Consequently, we propose that a moving node can create a P2P group by sending beacon frames on an individual channel, making its network discoverable by other devices. Legacy clients who perform a Wi-Fi scan can connect to a P2P GO, provided that they implement the required security mechanisms. This way, legacy devices see the P2P group owner as a traditional AP [26].

Both Bluetooth and Wi-Fi Direct traces had the same input parameters shown in Table 2. The output parameters for the Wi-Fi Direct trace, taking into consideration the transfer speed, are presented in Table 4.

**Figure 7:**
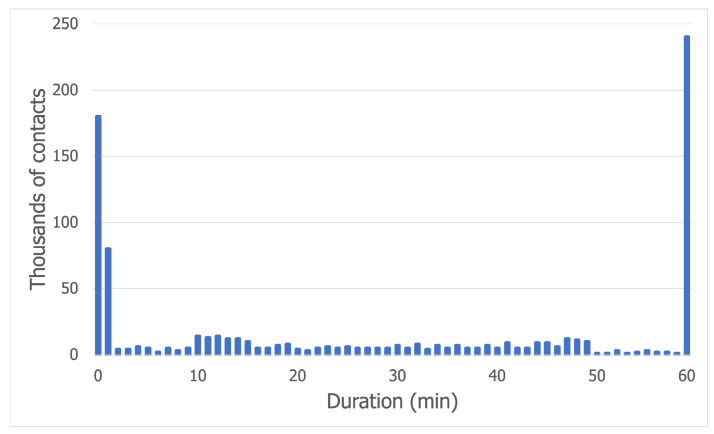
Contact duration distribution for the Wi-Fi Direct trace.

Figure 7, which shows the contact duration distribution for the Wi-Fi Direct trace, displays a trend very similar to the one in Figure 5, the only exception being that there are more long contacts than short-lived contacts. This observation can be explained by the behavior of AP nodes, which aim to preserve battery life. As these nodes alternate between the on and off states in 5-min intervals, the gap is caused by the 5 min when the AP is off.

#### 5.2.3. Transfer Speed

As mentioned in Section 3.2, the transfer speeds for Bluetooth and Wi-Fi Direct are 2 Mbps and 250 Mbps, respectively. This is a parameter of paramount importance if we want to achieve a realistic behavior. Assuming the message size to be 100 KB, this means that we can send 2.5 messages per second using Bluetooth, and 300 messages per second with Wi-Fi Direct.

### 5.3. Results

The results will be illustrated using the metrics presented in Section 5.1: delivery rate, overhead, delivery delay, and hop count. The two traces presented in Section 5.2 were used in order to demonstrate the performance of our opportunistic algorithms compared to the other algorithms tested. All simulations were performed using the MobEmu simulator [14] and the proposed festival mobility model. For each experiment, we performed five simulation runs with different random number generator seeds, and we show the average values in each chart. The rest of the simulation details are presented in Table 2, Table 3 and Table 4.

#### 5.3.1. Bluetooth Trace

**Figure 8:**
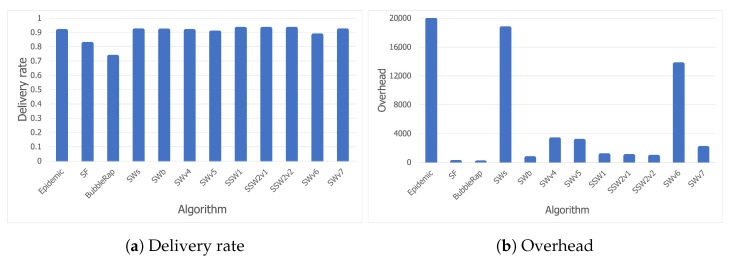
Delivery rate and overhead for the Bluetooth trace.

Figure 8a shows the delivery rates obtained by the analyzed algorithms for the Bluetooth trace. While most of the solutions tested have high delivery rate values in general, SF and Bubble Rap did not reach peak performance. SF proves that using last-encounter timestamps in a concert scenario is not a good strategy due to the lack of structure or repeating patterns. A node may choose another node as a relay for a message, even though it had recently encountered the message destination by chance and their paths are not likely to cross again. Moreover, the mechanism Bubble Rap uses for community detection is not reliable in the studied scenario. A node *A* is accepted as a member of the local community of another node *B*, provided that they have been in contact for a period of time which exceeds a threshold, or the familiarity set of node *A* contains enough community members of node *B*. Although this may work in most cases, a crowded event like a concert can mislead the algorithm to establish communities between nodes that have been staying next to each other, but have no social relationship in real life. There is a good potential for improving Bubble Rap with this knowledge, and we plan on doing so as future work.

As expected and as shown in Figure 8b, Epidemic has the highest overhead (due to its flooding-based mechanism), followed closely by SWs, whereas SWv6 might have too many constraints which prevent it from delivering the messages efficiently. Figure 8b narrows down our search for the most suitable opportunistic algorithm to be deployed at a music festival to three candidates: SWb, SSW1, and SSW2. For SSW2, we have performed the tests with two threshold values for centrality, each value corresponding to a version of the algorithm in the charts. SSW2v1 used a threshold of 50, while SSW2v2 used 100.

**Figure 9:**
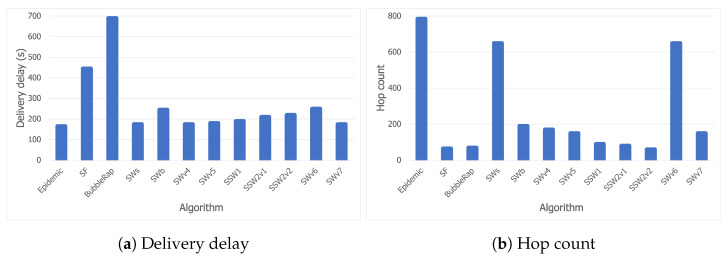
Delivery delay and hop count for the Bluetooth trace.

Figure 9a shows that, although there are other protocols with better delivery delay values than our selected algorithms, high overheads or low delivery rates prevent them from being considered. SWv5 has a comparable delivery rate and delivery delay to SSW1, but its more than double overhead disqualifies it from being taken into consideration. Furthermore, all three candidates have delays below 5 min, which should be considered an acceptable time in a delay-tolerant network.

Finally, Figure 9b shows that SWb has a lower hop count than the other two candidates (SSW1 and SSW2), aside from its slightly lower overhead. However, we have considered delivery delay to be the most essential criterion, so we can conclude that all three algorithms perform well in a festival scenario. While SWb may be the better choice if we want optimal resource usage, SSW1 and SSW2 will lead to lower delivery delay values with an insignificant extra cost.

#### 5.3.2. Wi-Fi Direct Trace

**Figure 10:**
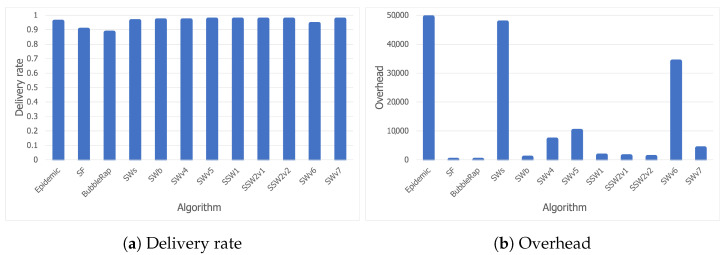
Delivery rate and overhead for the Wi-Fi Direct trace.

**Figure 11:**
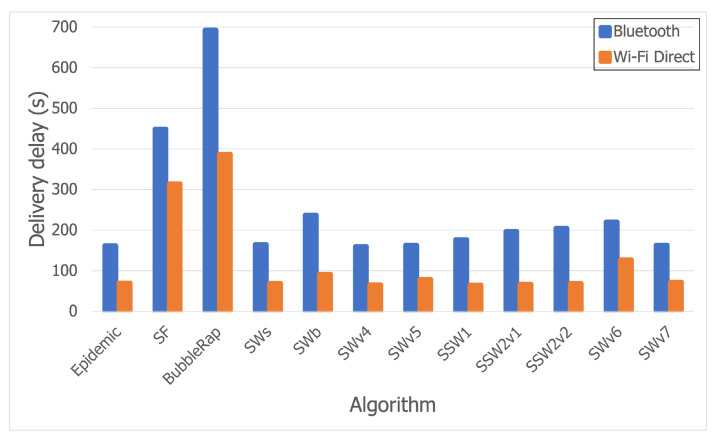
Delivery delay comparison between the Bluetooth and Wi-Fi Direct traces.

The results for the Wi-Fi Direct trace entirely support the conclusions stated in the Section 5.3.1, as Figure 10a,b shows that the same trends are followed. What immediately stands out are the improved delivery delay values for all the algorithms. In most cases, the delivery delay was reduced by more than half when combing Bluetooth with Wi-Fi Direct, as shown in Figure 11.

#### 5.3.3. Discussion

**Figure 12:**
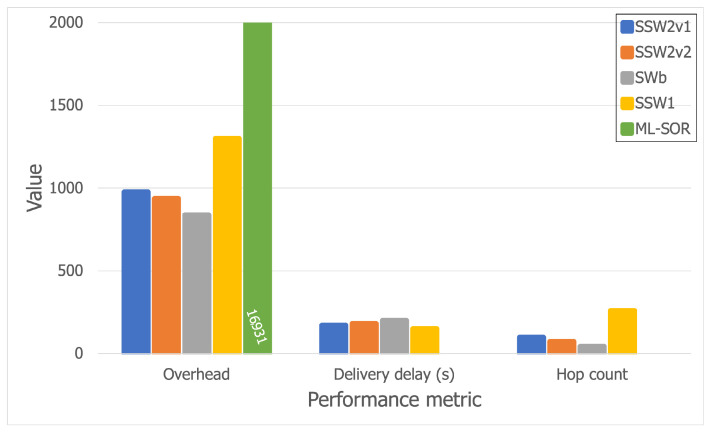
Results for the larger Bluetooth trace.

**Figure 13:**
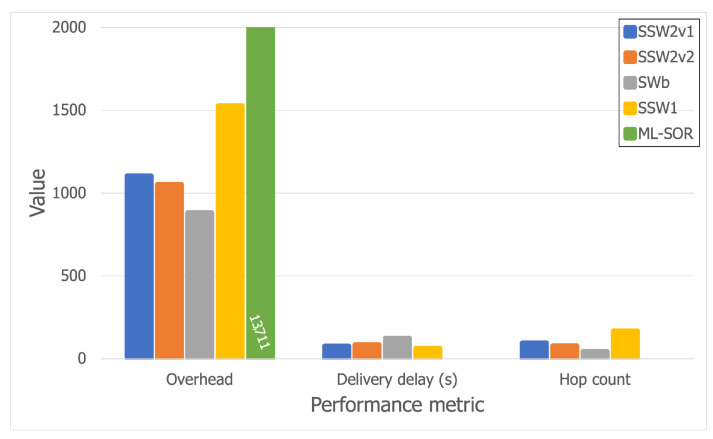
Results for the larger Wi-Fi Direct trace.

The best-performing algorithms, as stated previously, were SWb, SSW1, SSW2v1, and SSW2v2. In order to study their scalability and have a better understanding of their behavior, we further tested them using a larger simulation area of 40 × 40 m^2^. The results are thus shown in Figure 12 and Figure 13.

Furthermore, we also analyzed ML-SOR [27], which is a more advanced opportunistic routing and dissemination solution which assumes that there are multiple layers for device interaction, such as social connections, interests, etc. Based on these layers, ML-SOR nodes apply a utility function to each message upon a contact, in order to decide which message is forwarded to which encountered node. The solution thus uses multi-layer context information in order to improve the routing process. We chose this algorithm because it is a recent one that has been proven to have good results for various use cases, but was not tested in a festival scenario.

Thus, Figure 12 and Figure 13 show that all Spray-and-Wait-based algorithms scale relatively well, except for SSW1, which has a more dramatic overhead increase, from 1300 (on the larger Bluetooth trace) to 1527 (on the larger Wi-Fi Direct trace), but which is compensated by a decreased hop count. Moreover, while the hop count stays almost constant for SSWb, SSW2v1, and SSW2v2, SSW1 performs better when Wi-Fi Direct is used, as the hop count drops from 260 to 168. Furthermore, it can be observed that ML-SOR does not behave well at all in terms of overhead, which is far larger than that of any of the other algorithms.

Aside from this, ML-SOR does not manage to deliver a single message in this scenario, having a delivery rate of 0 (which is why the delivery delay and hop count are not depicted in Figure 12 and Figure 13, since they are only computed when messages reach their destinations). This shows that a solution such as ML-SOR might not be suitable for a festival scenario out of the box, mainly because of the density of the network and the large number of contacts in a short period of time. From our analysis, it appears that messages tend to bounce around between nodes in small areas, and thus are not able to move towards their destinations, which might be located farther. We plan to further extend this analysis and even modify ML-SOR to adapt it to a festival scenario.

Looking at the results, it can be observed that smaller delivery delay values can be accomplished at the price of higher overheads. Even so, there is a small difference in terms of overheads between SWb and SSW2, while the latter achieves better delivery delay values by far.

## 6. Conclusions and Future Work

In this paper, we introduced opportunistic routing in a scenario pertaining to a music festival, which is defined by a small space, a high number of participants, and a large density. In such a use case, congestion problems may easily arise due to the fact that people simultaneously connect to mobile broadband or Wi-Fi access points.

In order to assess the feasibility of opportunistic solutions in a festival scenario, we first analyzed existing mobility traces for such events and proposed a mobility model called FMM, which behaves similarly to real-life human behavior, considering the crowd’s social structure. We have also designed a more realistic way of selecting the destination of a message, since some works leave this decision to a random event generator.

Then, we analyzed existing opportunistic solutions and proposed and implemented our own custom opportunistic routing algorithms. We analyzed their performance on the FMM mobility model, and the results obtained indicated three potential candidates: binary Spray-and-Wait and our two custom opportunistic algorithms, Social Spray-and-Wait-1 and Social Spray-and-Wait 2. Moreover, this work further emphasizes two design decisions one such algorithm may implement, which can lead to high performance: a mechanism that bounds the number of message copies in order to avoid a resource-consuming flood-based forwarding scheme [4]; and incorporating social metrics [21] in the forwarding decision, which can enhance an algorithm and achieve lower delivery delay values.

Another goal was to study the impact of Bluetooth and Wi-Fi Direct on performance. The findings show that all three algorithms perform better when combing Wi-Fi Direct with Bluetooth, mostly due to the increased speed of transfer and communication range of Wi-Fi Direct.

In terms of future work, we would like to implement a more complex battery consumption mechanism, extend the Festival Mobility Model to be able to produce larger maps with more stages, and modify the speed of transfer according to the number of simultaneous contacts. Furthermore, we wish to extend our analysis of both the FMM mobility model, as well as of the two proposed routing solutions, in order to have a better view of the requirements and behavior of a music festival scenario.

In conclusion, using opportunistic networks for end-to-end message delivery at music festivals is a viable option with promising performance results. Where the traditional network infrastructure fails, opportunistic networks may be the solution we are looking for.

## Figures and Tables

**Table 1:** Opportunistic algorithm versions.

Name	Spray Phase	Wait Phase
SWv1	SWb	FC1
SWv2	SWb	FC2
SWv3	SWb	FC3
SWv4	if FC3 {SWb} else {SWs}	SW wait
SWv5	if FC2 {SWb} else {SWs}	SW wait
SSW1	if (FC2 or FC3) {SWb} else {SWs}	SW wait
SSW2	if (FC2 or FC3) {SWb} elseif FC4 {SWb} else {SWs}	SW wait
SWv6	if (FC2 and FC3) {SWb} else if FC1 {SWs}	SW wait
SWv7	if FC3 {SWb} else if FC2 {SWs}	SW wait

**Table 2:** Input parameters for the Bluetooth and Wi-Fi traces.

Parameter	Value
Area	30 × 20 m^2^
Protocol	Bluetooth
Bluetooth range	5 m
Wi-Fi Direct range	30 m
Message size	100 KB
Data memory	5000 messages
Trace duration	1 h
Group size	5

**Table 3:** Bluetooth trace output parameters.

Parameter	Value
Nodes	1700
Contacts	839,680
Messages	8282
Maximum neighbors	7

**Table 4:** Wi-Fi Direct trace output parameters.

Parameter	Value
Nodes	1700
Contacts	683,945
Messages	8282
Maximum neighbors	7

## Data Availability

Not applicable.
